# Correction: Know your enemy: Application of ATR-FTIR spectroscopy to invasive species control

**DOI:** 10.1371/journal.pone.0271370

**Published:** 2022-07-08

**Authors:** Claire Anne Holden, John Paul Bailey, Jane Elizabeth Taylor, Frank Martin, Paul Beckett, Martin McAinsh

The fourth author’s name is spelled incorrectly. The correct name is: Francis L Martin. The correct citation is: Holden CA, Bailey JP, Taylor JE, Martin FL, Beckett P, McAinsh M (2022) Know your enemy: Application of ATR-FTIR spectroscopy to invasive species control. PLoS ONE 17(1): e0261742. https://doi.org/10.1371/journal.pone.0261742
