# Supplementary figures and images for: Know your enemy: Application of ATR-FTIR spectroscopy to invasive species control
Source: PLoS One. 2022 Jan 7;17(1):e0261742. doi: 10.1371/journal.pone.0261742 (PMC8740966; doi:10.1371/journal.pone.0261742)

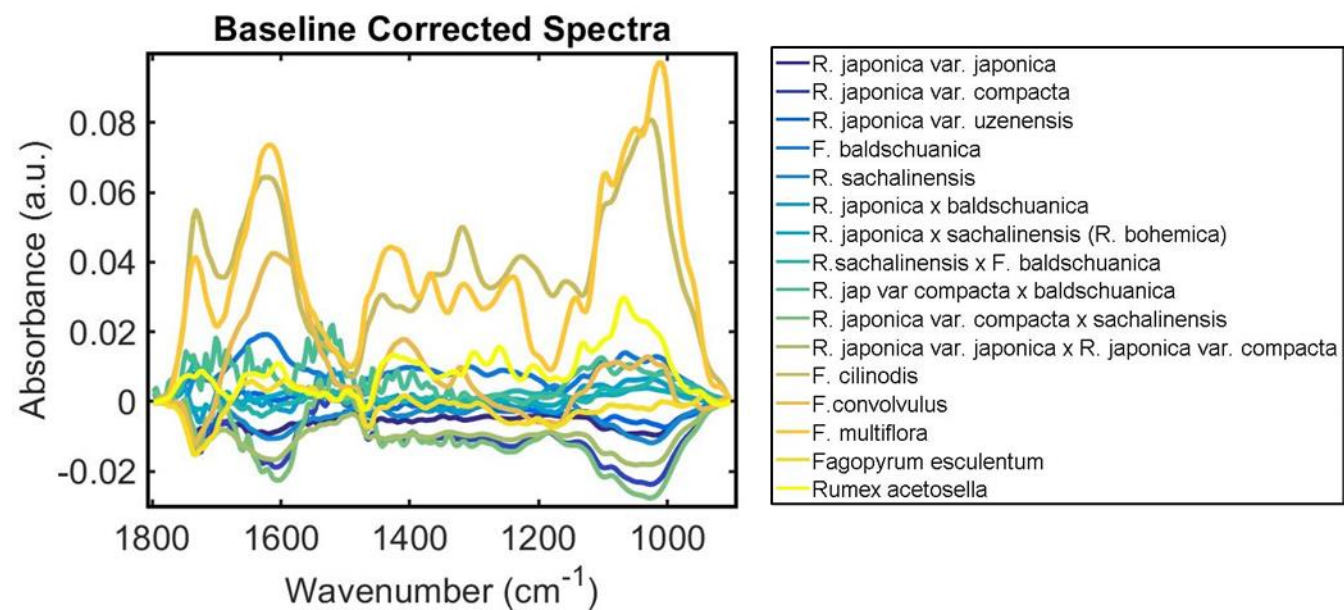

**S1 Figure:** Baseline corrected spectra

Supplement: S1 Fig — (PDF) [file pone.0261742.s001.pdf]

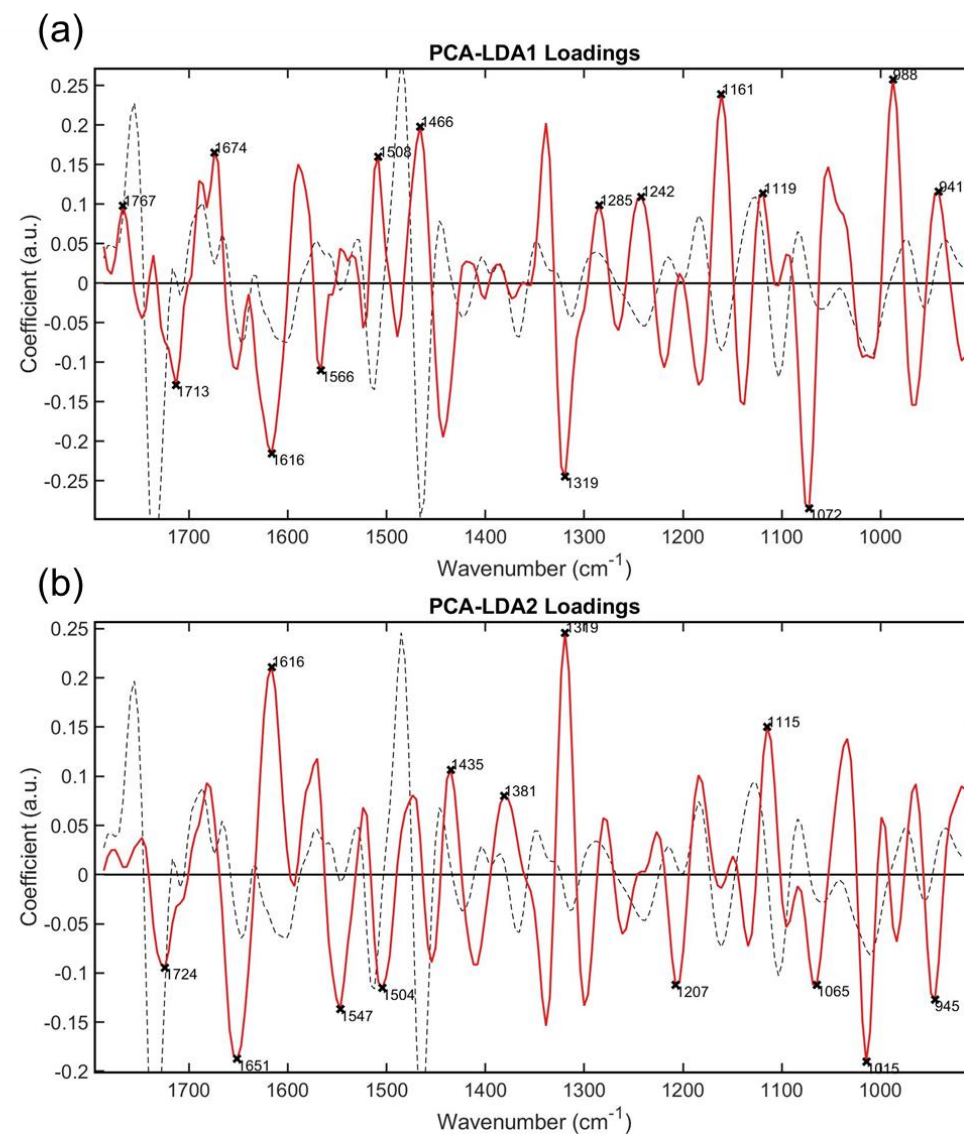

**S3 Figure:** PCA-LDA loadings graphs and key wavenumbers used for HCA analysis

Supplement: S3 Fig — (PDF) [file pone.0261742.s003.pdf]
