# Supplementary material for: Know your enemy: Application of ATR-FTIR spectroscopy to invasive species control
Source: PLoS One. 2022 Jan 7;17(1):e0261742. doi: 10.1371/journal.pone.0261742 (PMC8740966; doi:10.1371/journal.pone.0261742)
Supplement: S2 Fig — (a) PCA scores plot, (b) LDA 2D scatter plot, (c) SVM scores plot and (d) SVM classification table of fingerprint spectra grouped by leaf surface: upper (blue) and lower (yellow) leaf surfaces. (PDF) [file pone.0261742.s002.pdf]

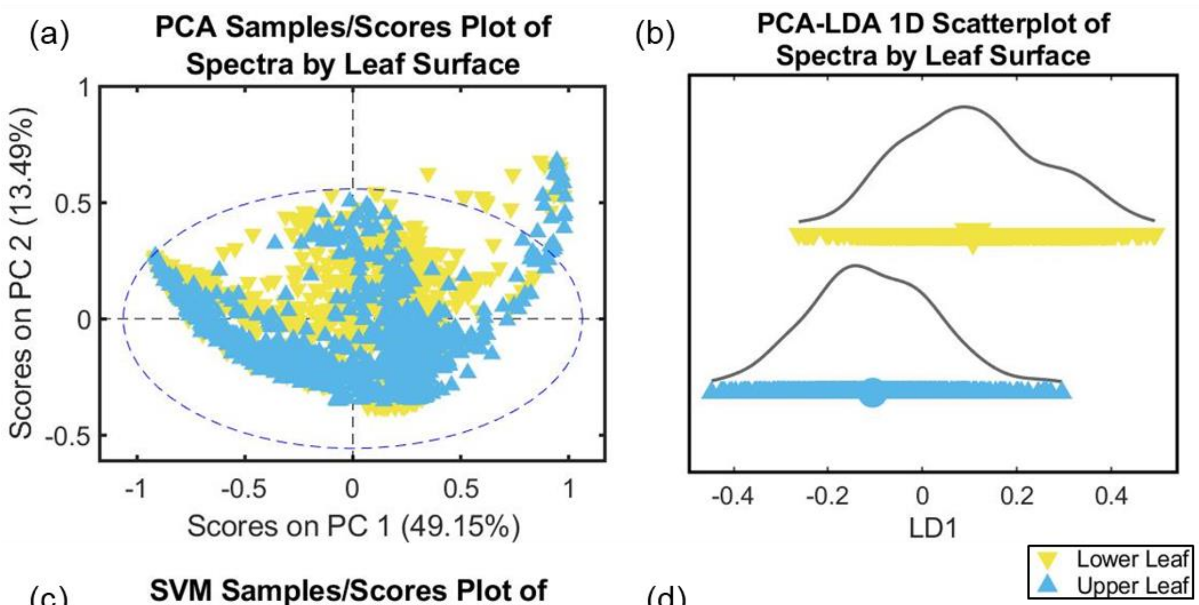

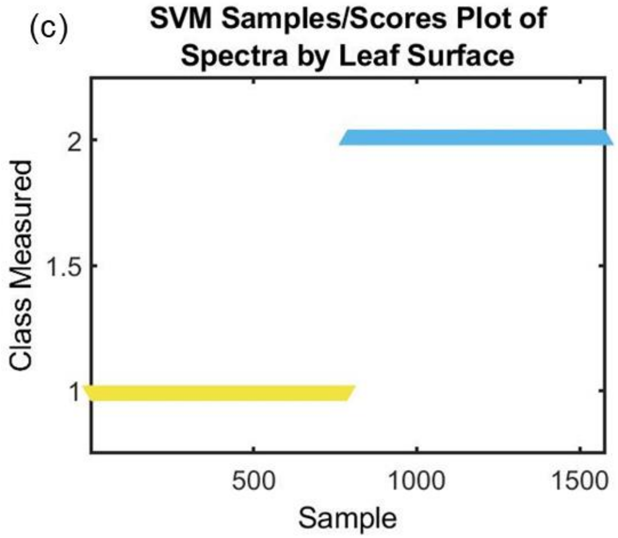

(d)

	% Accuracy	% Sensitivity	% Specificity
**Lower**	98.4	98.5	98.2
**Upper**	98.4	98.2	98.5

S2 Figure. (a) PCA scores plot, (b) LDA 2D scatter plot, (c) SVM scores plot and (d) SVM classification table of fingerprint spectra grouped by leaf surface: upper (blue) and lower (yellow) leaf surfaces.
