# Supplementary material for: Know your enemy: Application of ATR-FTIR spectroscopy to invasive species control
Source: PLoS One. 2022 Jan 7;17(1):e0261742. doi: 10.1371/journal.pone.0261742 (PMC8740966; doi:10.1371/journal.pone.0261742)
Supplement: S4 Fig — Scanning Electron Microscope images of the lower epidermis of (a) Reynoutira japonica and (b) Reynoutria sachalinensis. (PDF) [file pone.0261742.s004.pdf]

(a) **Lower epidermis of *R. japonica***

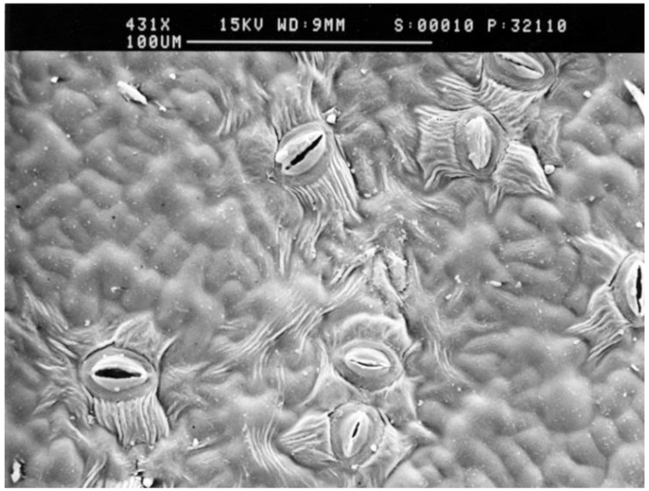

(b) **Lower epidermis of *R. sachalinensis***

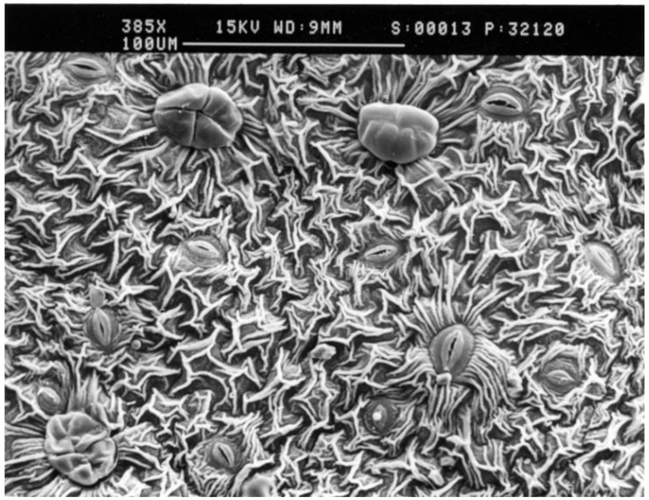

S4 Figure: Scanning Electron Microscope images of the lower epidermis of **(a)** *Reynoutria japonica* and **(b)** *Reynoutria sachalinensis*
