# Supplementary material for: Know your enemy: Application of ATR-FTIR spectroscopy to invasive species control
Source: PLoS One. 2022 Jan 7;17(1):e0261742. doi: 10.1371/journal.pone.0261742 (PMC8740966; doi:10.1371/journal.pone.0261742)
Supplement: S5 Fig — See attached MATLAB file to zoom. (PDF) [file pone.0261742.s005.pdf]

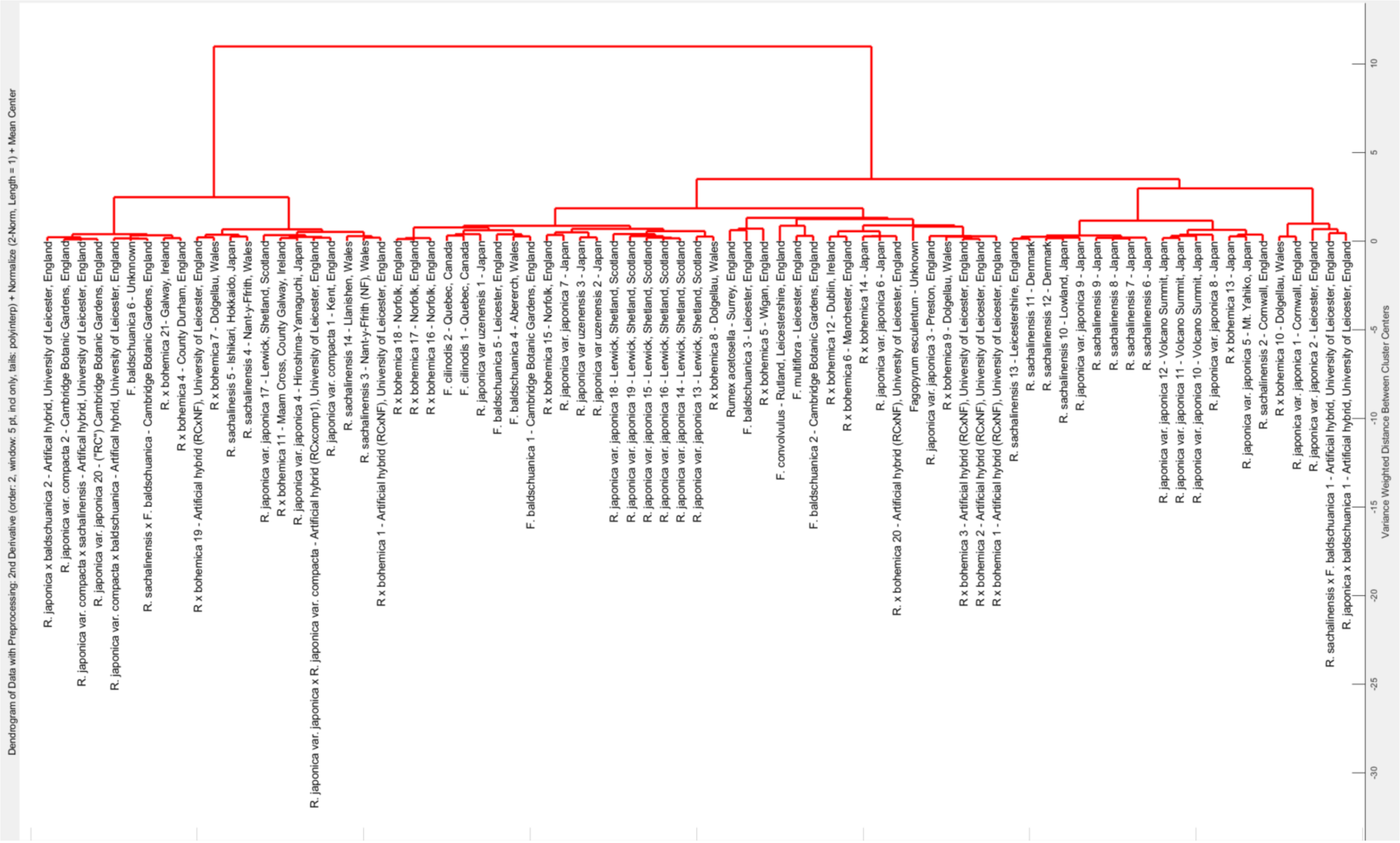

S5 Figure: HCA analysis using fingerprint region (1800-900 cm^{-1}) for each sample with species and location information. See attached MATLAB file to zoom in.
