# Supplementary material for: Know your enemy: Application of ATR-FTIR spectroscopy to invasive species control
Source: PLoS One. 2022 Jan 7;17(1):e0261742. doi: 10.1371/journal.pone.0261742 (PMC8740966; doi:10.1371/journal.pone.0261742)
Supplement: S1 Table — (PDF) [file pone.0261742.s007.pdf]

S1 Table: Herbarium sample information

P Number	F Number	Country	Location	Grid Reference	Species	Chromosome Number	Collected By	Collection Date	Additional Info
**P942**		Japan	Lowland		*R. sachalinensis*	2n=44			
**P918**		Japan	Lowland		*R. sachalinensis*	2n=44			
**P920**		Japan			*R. sachalinensis*	2n=44			
**P994**		Japan	Volcano Summit		*F. japonica* var. *japonica*	2n=44			
**P995**		Japan	Volcano Summit		*F. japonica* var. *japonica*	2n=cc44 (circa)			
**P991**		Japan	Volcano Summit		*F. japonica* var. *japonica*	2n=44			
**P1051**		Japan			*F. x bohemica*	2n=44			
**P1052**		Japan			*F. x bohemica*	2n=44			
**P939**		Japan			*F. japonica* var. *japonica*	2n=66			
**P940**		Japan			*F. japonica* var. *japonica*	2n=66			
**P981**		Japan	Mt. Yahiko		*F. japonica* var. *japonica*	2n=44			206 cm giant tetraploid
**P977**		Japan			*F. japonica* var. *uzenensis*	2n=88			hairy knotweed
**P978**		Japan			*F. japonica* var. *uzenensis*	2n=cc88			hairy knotweed

**P980**		Japan			*F. japonica* var. *uzenensis*	2n=88			hairy knotweed
**P987**		Japan			*F. japonica* var. *japonica*	2n=88			Hirokuta city
**P986**		Japan			*F. japonica* var. *japonica*	2n=44			160 cm tall
**P976**		Japan			*R. sachalinensis*	2n=44			Coastline
**P975**		Japan			*R. sachalinensis*	2n=44			Coastline
		England	Cambridge Uni Botanic Gardens		*F. baldschuanica*	2n=20			"labelled *baldschuanica*"
		England	Cambridge Uni Botanic Gardens		*F. baldschuanica*	2n=20		04/08/1996	"labelled *aubertii*"
		England	Hinckley Road Leicester		*F. baldschuanica*	2n=20		05/10/1985	
		UK	The Barn Aberech		*F. baldschuanica*	No chromosome data			
		UK	Tim Rich's Garden		*F. baldschuanica*	No chromosome data		1983	
	F461a	Wales	Mawddach, downstream from Dolgellau bridge	GR 23/720.183 vc 48					Group A Leaf 1
		Wales	Mawddach, downstream from	GR 23/720.183 vc 49					Group A Leaf 2

			Dolgellau bridge						
		Wales	Mawddach, downstream from Dolgellau bridge	GR 23/720.183 vc 50					Group B Leaf 1
		Wales	Mawddach, downstream from Dolgellau bridge	GR 23/720.183 vc 51					Group B Leaf 2
		Japan	Hokkoido Ishikori		*R. sachalinensis*	2n=44	D. Ratcliffe + T. Rich		
	F86	Ireland	West end of L, East of Maam Cross		*F. x bohemica*		JP Bailey	12/07/1995	
	F586	Ireland	Booterstown Railway, Dulbin		*F. x bohemica*				
	F289	Ireland	West Galway, Coast road north of roundstone	GR 02/726.424	*F. x bohemica*	2n=66	Ann Conolly	Sep-79	
	F637	England	Haigh Country Park, Wigan. Site B	GR 600.078	*F. x bohemica*		Blackhall	1995	
	F638	England	Grove Park Timperly. Site A	GR/ 783.874	*F. x bohemica*		B. Blackhall	1995	

	F254	England	South Wylam, County Durham		*F. x bohemica*	2n=44	Ann Conolly		Between Railway and River Tyne
**P1092**		Japan	Hiroshima-Yamaguchi		*F. japonica* var. *japonica*	2n=44			3m tall, giant tetraploid
	F214		ex. Platt Bracken Hill		*F. japonica* var. *japonica x F. japonica* var. *compacta*		JP Bailey	09/10/1984	
	F563				*F. japonica* var. *compacta*		JP Bailey	26/09/1984	Artificial Hybrid: *F. japonica* var. *japonica* (race course) x *F. japonica* var. *compacta* (Comp 1)
	F282				*R. jap x R. sach*	2n=66		19/09/1984	Artificial hybrid: *R. jap* (race course) x *R. sachalinensis* (NF)
	F317	Wales	Howey, S of Llandrindod Wells	GR 32/0..5..	*R. sach*		Ann Conolly	Aug-62	*R. sachalinensis* "HOWEY"
	F512				*F. x bohemica*		JP Bailey	14/09/1987	RC x NF3 - leaf 1
	F350				*R. sachalinensis*		Ann Conolly	05/09/1977	NF - leaf 1
	F350				*R. sachalinensis*		Ann Conolly	05/09/1977	NF - leaf 2

	F512				*F. x bohemica*		JP Bailey	14/09/1987	RC x NF3 - leaf 2
	F512				*F. x bohemica*		JP Bailey	14/09/1987	RC x NF3 - leaf 3
	F186	England	Penwaltham Church, Preston		*F. japonica* var. *japonica*				
	F168	England	Mevagissey, East Cornwall		*F. japonica* var. *japonica*				
	F198	England	Highcross + Claybridge Magna. Leicester		*F. japonica* var. *japonica*				
	F558a				*R. sachalinensis x F. bald*	2n=32			Grown from seed collected from Cheshunt
	F2205				*R. jap x F.bald*	2n=54			
		England	Penwith, Cornwall	GR 10/4465.2645	*R. sachalinensis*				
	P140a	Wales	Buryas Bridge		*Sachalinensis x F. baldschuanica*				Open poll seed from P160 Llanishan (*sach*). Hybrid: Giant knotweed crossed with Russian Vine
	P1190	England	Cambridge Uni Botanic Gardens		*F. japonica* var. *compacta*				(spec F2053)
	F2148				*F. baldschuanica*	2n = 20			

	F2206				*F. japonica* var. *japonica* *x* *baldschuanica*				Artificially crossed at Leicester University by Dr. John Bailey, 2n= 54
	F2209				*F. japonica* var. *compacta* *x* *baldschuanica*	2n= 32			Artificial hybrid
	F87		Collected from race course cultivated at botanic gardens		*F. japonica* var. *japonica*				
	F236				*F. japonica* var. *compacta* *x male sachalinensis*				Compact variety of Japanese knotweed crossed with a male Giant knotweed from Nat Y Frith
	F518				*F. japonica* var. *japonica* *x* *sachalinensis*				*F. japonica* var. *japonica* from race course x male sachalinensis from Nat Y Frith, artificial hybrid P75D

	F493	Wales	Llanishan, Wales		*R. sachalinensis*				
					Fagopyrum esculentum			14/07/1930	
		England	Wisley Common, Surrey		*Rumex acetosolla*			10/05/1964	
	F401				*F. multiflora*		J. Bailey	Sep-85	Cultivated LJR
		England	Bloody Oaks Quarry, Rutland		*F. convolvulus*		EK Horwood	11/10/1951	
	F409	Canada	Champion Lookout, Quebec, Canada		*F. cilinodis*			05/08/1987	
