# Supplementary material for: Know your enemy: Application of ATR-FTIR spectroscopy to invasive species control
Source: PLoS One. 2022 Jan 7;17(1):e0261742. doi: 10.1371/journal.pone.0261742 (PMC8740966; doi:10.1371/journal.pone.0261742)
Supplement: S2 Table — (PDF) [file pone.0261742.s008.pdf]

S2 Table: SVM parameters

SVM Classification	Cost	Gamma (γ)	Number of support vectors (N_{SV})
**Sample type of closely related species, hybrids, and varieties**	100	1	613
**Upper and lower surfaces**	31.6228	3.1623	327
**Geographical Areas**	100	0.1	111
