# Supplementary material for: Know your enemy: Application of ATR-FTIR spectroscopy to invasive species control
Source: PLoS One. 2022 Jan 7;17(1):e0261742. doi: 10.1371/journal.pone.0261742 (PMC8740966; doi:10.1371/journal.pone.0261742)
Supplement: S3 Table — (PDF) [file pone.0261742.s009.pdf]

S3 Table: Quality parameters (accuracy, sensitivity, and specificity) for spectral classification based on sample type of closely related species, hybrids, and varieties by PCA-LDA

PCA-LDA	% Accuracy	% Sensitivity	% Specificity
R. japonica* var. *japonica	62.87	29.90	83.41
R. japonica* var. *compacta	86.59	12.50	90.74
R. japonica* var. *uzenensis	86.93	63.33	88.99
F. baldschuanica	89.07	46.22	97.39
R. sachalinensis	74.43	75.00	74.16
R. japonica x baldschuanica	92.35	0.00	97.75
R. japonica x sachalinensis (R. x bohemica)	60.65	19.71	87.00
R. sachalinensis x F. baldschuanica	83.59	47.50	85.54
R. japonica* var. *compacta x baldschuanica	97.02	47.62	98.62
R. japonica* var. *compacta x sachalinensis	86.70	30.00	88.25
R. japonica* var. *japonica x R. japonica* var. *compacta	80.10	0.00	82.12
F. cilinodis	95.04	70.00	96.59
F. convolvulus	100.00	100.00	100.00
F. multiflora	96.88	100.00	96.78
Fagopyrum esculentum	100.00	100.00	100.00
Rumex acetosella	96.74	95.00	96.79
**Average**	**86.81**	**52.30**	**91.51**
