# Supplementary material for: Know your enemy: Application of ATR-FTIR spectroscopy to invasive species control
Source: PLoS One. 2022 Jan 7;17(1):e0261742. doi: 10.1371/journal.pone.0261742 (PMC8740966; doi:10.1371/journal.pone.0261742)
Supplement: S4 Table — (PDF) [file pone.0261742.s010.pdf]

S4 Table: Quality parameters for spectral classification based on sample type of closely related species, hybrids, and varieties by SVM.

Class	% Accuracy	% Sensitivity	% Specificity
R. japonica **var.** ***japonica***	99	98	99
R. japonica **var.** ***compacta***	96	93	100
R. japonica **var.** ***uzenensis***	100	100	100
F. baldschuanica	99	98	100
R. sachalinensis	99	98	100
R. japonica x baldschuanica	97	95	100
R. japonica x sachalinensis (R. x bohemica)	98	97	99
R. sachalinensis x F. baldschuanica	99	98	100
R. japonica **var.** ***compacta x baldschuanica***	98	95	100
R. japonica **var.** ***compacta x sachalinensis***	100	100	100
R. japonica **var.** ***japonica x R. japonica*** **var.** ***compacta***	100	100	100
**Average**	**99**	**97**	**100**
