# Supplementary material for: Know your enemy: Application of ATR-FTIR spectroscopy to invasive species control
Source: PLoS One. 2022 Jan 7;17(1):e0261742. doi: 10.1371/journal.pone.0261742 (PMC8740966; doi:10.1371/journal.pone.0261742)
Supplement: S5 Table — (PDF) [file pone.0261742.s011.pdf]

S5 Table: Quality parameters (accuracy, sensitivity, and specificity) for spectral classification of R. japonica var. japonica based on geographical location by PCA-LDA

PCA-LDA	% Accuracy	% Sensitivity	% Specificity
**England**	86.81	64.56	92.67
**Scotland**	92.16	95.00	90.32
**Japan**	84.58	81.01	87.62
**Average**	**87.85**	**80.19**	**90.20**
